# Generalized Venous Prominence on Susceptibility-Weighted Imaging Correlates With Global Cerebral Blood Flow Decline

**DOI:** 10.7759/cureus.56272

**Published:** 2024-03-16

**Authors:** Emilian Kalchev

**Affiliations:** 1 Diagnostic Imaging, University Hospital St. Marina, Varna, BGR

**Keywords:** susceptibility-weighted imaging, neurovascular disorders, arterial spin labeling mri, cerebral veins, cerebral perfusion, cerebral blood flow

## Abstract

Objective

This study investigated the global correlation between cerebral blood flow (CBF) decline and increased venous prominence, utilizing arterial spin labeling (ASL) and susceptibility-weighted imaging (SWI) MRI techniques.

Methods

The study was conducted at the Department of Diagnostic Imaging, St. Marina University Hospital, Varna, Bulgaria. Through a retrospective analysis, we examined data from 115 patients undergoing neurological assessment. CBF decline was assessed through ASL MRI, while global venous visibility was evaluated using SWI MRI.

Results

The analysis revealed a significant positive correlation between CBF decline and venous prominence (Spearman's rho = 0.261, p = 0.005), indicating a systemic interaction between cerebral perfusion and the venous system. Logistic regression further underscored CBF decline as a significant predictive factor for increased venous visibility (odds ratio (OR) = 1.690, p = 0.004). The assessments' high inter-rater reliability (Cohen's kappa = 0.82) supports the consistency and validity of our findings.

Conclusion

The integration of ASL and SWI MRI provides critical insights into cerebral hemodynamics, emphasizing the significance of these imaging modalities in both neurovascular research and clinical practice. Our findings suggest a systemic relationship between CBF decline and venous system alterations, underscoring the potential for these techniques to enhance our understanding of neurovascular disorders. Future studies should pursue longitudinal and quantitative analyses to deepen our comprehension of these relationships and their clinical implications.

## Introduction

Cerebral blood flow (CBF) is foundational to brain function, facilitating the delivery of oxygen and nutrients while removing metabolic wastes [1]. Arterial spin labeling (ASL) MRI represents a significant advancement in the non-invasive quantification of CBF [2,3,4]. By using magnetically labeled arterial blood as an endogenous tracer, ASL MRI allows for detailed cerebral perfusion mapping without the risks associated with radioactive tracers. This method enables safe, repeated assessments of cerebral perfusion, offering a valuable tool for diagnosing and managing neurological disorders, without the risks associated with contrast media or radioactive tracers [5].

Susceptibility-weighted imaging (SWI) MRI, utilizing the magnetic susceptibility differences between tissues, offers unparalleled visualization of venous structures, microbleeds, and other vascular abnormalities within the brain [6,7,8]. This advanced technique particularly excels in detecting the subtle contrasts caused by deoxygenated blood in veins, making venous networks distinctly visible against the surrounding brain tissue. The detailed imagery provided by SWI MRI is instrumental in diagnosing and understanding a wide array of neurological conditions, from identifying microvascular changes to assessing the extent of traumatic brain injuries or strokes. Its ability to reveal the intricate venous architecture complements the cerebral perfusion data obtained from ASL MRI, thereby enriching our understanding of the brain's vascular system as a whole [9].

The independent value of ASL and SWI MRI in studying cerebral arterial inflow and venous structures, respectively, raises intriguing questions about the relationship between cerebral perfusion and venous visibility. Investigating how alterations in CBF may correlate with changes in the venous system offers a novel avenue for understanding the complex interplay within cerebral hemodynamics.

Accordingly, this study aims to examine the global correlation between CBF decline, as measured by ASL MRI, and changes in venous prominence, as detected through SWI MRI. By exploring the potential linkage between these two distinct but essential aspects of cerebral hemodynamics, we hope to uncover insights that could impact the diagnosis, monitoring, and treatment of neurovascular disorders.

## Materials and methods

The study was conducted at the Department of Diagnostic Imaging, St. Marina University Hospital, Varna, Bulgaria. We employed a retrospective design to explore the global correlation between CBF decline and venous visibility, utilizing data from patients who underwent both ASL and SWI MRI. The analysis was based on a cohort from the database of our department, focusing on scans performed between 2016 and 2020.

Participants

The cohort comprised 115 patients, selected based on specific inclusion and exclusion criteria. Inclusion criteria mandated that participants had undergone both ASL and SWI MRI as a part of their standard neurological assessment. Exclusion criteria were established to ensure the clarity and relevance of the data, excluding patients with conditions that significantly alter local cerebral hemodynamics, such as major acute stroke and large cerebral masses, or those who had undergone recent cerebral radiotherapy. To refine the focus on global cerebral changes, our exclusion criteria also omitted patients with asymmetrical venous prominence or CBF decline across hemispheres, indicative of localized pathology. In addition, scans of poor quality that precluded accurate evaluation were omitted. The resulting demographic spread included ages from 45 to 84 years, with a near-even distribution between genders, ensuring a diverse study population reflective of the general patient base undergoing neurological evaluation.

MRI techniques

The study utilized a comprehensive MRI protocol including T1-weighted imaging (T1WI), T2-weighted imaging (T2WI), fluid-attenuated inversion recovery (FLAIR), diffusion-weighted imaging (DWI), and SWI. SWI provided magnitude, phase, SWI, and minimum intensity projection (MIP) series, offering a detailed view of venous structures and microvascular changes. In addition, 3D pulsed ASL was employed for CBF assessment, focusing on qualitative evaluation. Imaging parameters were optimized across sequences to ensure high-quality, consistent data for analysis.

Data collection

**Figure 1:**
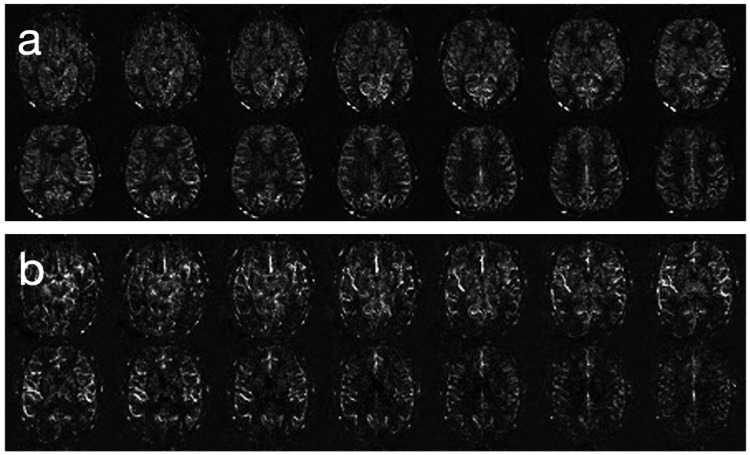
Comparative ASL CBF mosaic views illustrating varying degrees of perfusion decline a: ASL CBF mosaic view demonstrating mild global perfusion decline, categorized as ASL stage 1 on the ASL visual rating scale [10]. This image showcases subtle reductions in cerebral perfusion, indicative of the early stages of perfusion decline. b: ASL CBF mosaic view depicting more pronounced global perfusion decline, classified as ASL stage 3 according to the ASL visual rating scale [10]. The image highlights significant reductions in cerebral perfusion, representing advanced stages of global perfusion decline. ASL: arterial spin labeling, CBF: cerebral blood flow

Global CBF was quantified using an ASL visual rating scale [10], with scores ranging from 0 (indicating normal perfusion) to 4 (indicating severe perfusion decline), enabling a standardized evaluation of cerebral perfusion throughout the study population (Figure 1).

**Figure 2:**
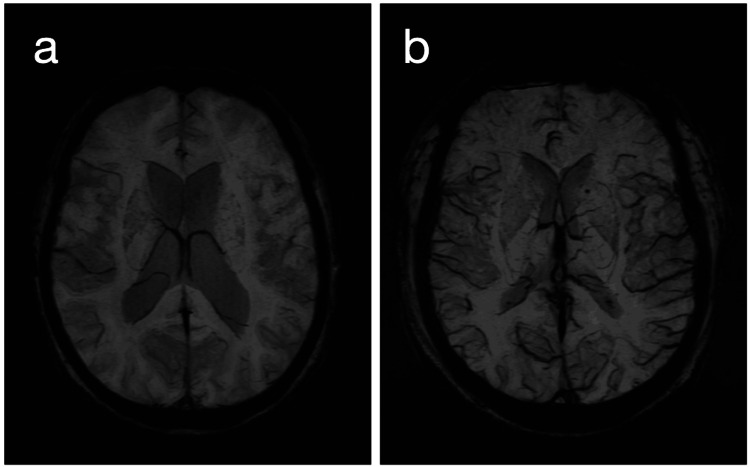
MIP SWI images demonstrating variations in cerebral venous prominence a: MIP SWI image without increased prominence of cerebral veins. This image represents a normal or baseline state, with cerebral veins appearing within expected visibility parameters, lacking signs of global prominence. b: MIP SWI image showing globally prominent cerebral veins. This image illustrates a condition where cerebral veins are distinctly more visible throughout the brain, indicative of increased venous prominence on a global scale. MIP: minimum intensity projection, SWI: susceptibility-weighted imaging

For venous visibility, SWI analysis, conducted on minimum intensity projection (MIP) images, encompassed a holistic assessment of the cerebral venous network, aiming to identify any instances of generalized venous prominence. This broader approach sought to capture variations in venous visibility across the entire brain, classifying them as either present or absent (Figure 2).

Two experienced radiologists independently reviewed the images, with any discrepancies resolved through consensus, ensuring the reliability and validity of the assessments.

Statistical analysis

Statistical analysis was performed using IBM SPSS Statistics for Windows, version 29 (released 2022; IBM Corp., Armonk, New York, United States). The relationship between CBF decline and venous visibility was assessed using Spearman's rho correlation coefficient to evaluate the strength and direction of the association. Logistic regression analysis was applied to explore predictive factors for venous visibility, incorporating CBF decline scores, age, and gender as independent variables. Inter-rater reliability for the qualitative assessment of venous visibility was quantified using Cohen's kappa coefficient, ensuring the consistency of visual evaluations between the two radiologists.

## Results

Population characteristics

Our study analyzed a cohort of 115 patients, encompassing a wide age range from 45 to 84 years, with a mean age of 62.48 years (standard deviation (SD) = 10.3). The gender distribution was 36% female and 64% male, reflecting a diverse demographic representative of the general population seeking neurological assessment (Table 1).

**Table 1:** Population descriptive statistics This table outlines the descriptive statistics for the study population's demographic and clinical variables. The average age of the participants is 62.48 years with a standard deviation indicating diversity in the age range. For gender, coded as 0 for males and 1 for females, the mean value suggests a higher proportion of males in the sample. The ASL stage shows an average of 1.32, reflecting a mild to moderate decline in cerebral blood flow. SWI imaging revealed prominent veins in approximately 34.8% of cases. ASL: arterial spin labeling, SWI: SWI: susceptibility-weighted imaging

	N	Mean	Std. deviation	Variance
Age	115	62.478	10.255	105.171
Gender	115	.357	.479	.229
ASL stage	115	1.322	1.255	1.575
SWI prominent veins	115	.348	.476	.227

Main findings

**Figure 3:**
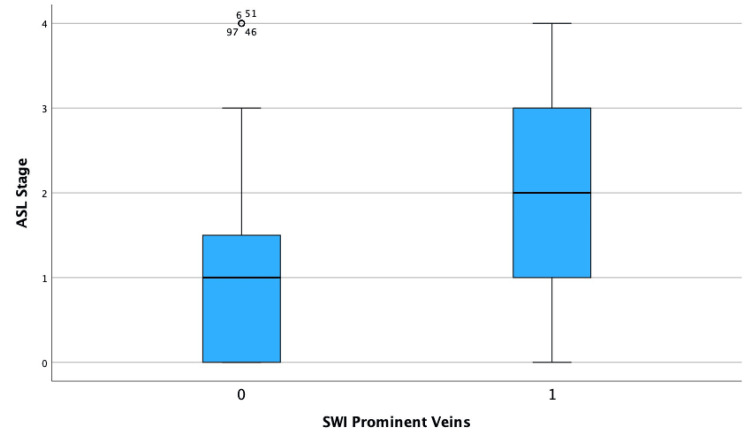
Box plot of ASL stages grouped by SWI visibility of prominent veins This figure displays a box plot comparing the distribution of ASL stages between patients with no visible prominent veins (SWI prominent veins = 0) and those with visible prominent veins (SWI prominent veins = 1). The central line in each box represents the median ASL stage, while the edges of the boxes correspond to the 25th and 75th percentiles, depicting the interquartile range. This visual comparison reveals a higher median ASL stage in patients with visible prominent veins, consistent with the findings of a positive correlation between the ASL stage and venous visibility. ASL: arterial spin labeling, SWI: susceptibility-weighted imaging

The investigation revealed a statistically significant correlation between global CBF decline, as assessed by the ASL visual rating scale, and increased visibility of venous structures on SWI MRI. The Spearman's rho correlation coefficient was calculated to be 0.261, indicating a positive relationship between CBF decline and venous visibility (p = 0.005) (Table 2). This result suggests that as global CBF decreases, the likelihood of observing increased venous prominence in the brain correspondingly rises (Figure 3).

**Table 2:** Spearman's rho correlation coefficients between study variables This table displays the Spearman's rho correlation coefficients indicating the strength and direction of associations between age, gender, ASL Stage, and the visibility of prominent veins as detected by SWI MRI. Significant correlations are highlighted: ASL stage shows a positive correlation with both age (rho = 0.225, p = 0.016) and SWI prominent veins (rho = 0.261, p = 0.005), suggesting that increases in ASL stage are associated with advancing age and greater visibility of venous structures. ASL: arterial spin labeling, SWI: susceptibility-weighted imaging

		Age	Gender	ASL stage	SWI prominent veins
Age	Correlation coefficient	1.000	0.042	0.225	-0.155
	Sig. (2-tailed)	.	0.657	0.016	0.098
Gender	Correlation coefficient	0.042	1.000	0.321	0.066
	Sig. (2-tailed)	0.657	.	<0.001	0.481
ASL stage	Correlation coefficient	0.225	0.321	1.000	0.261
	Sig. (2-tailed)	0.016	<0.001	.	0.005
SWI prominent veins	Correlation coefficient	-0.155	0.066	0.261	1.000
	Sig. (2-tailed)	0.098	0.481	0.005	.

Regression analysis

Logistic regression analysis further elucidated the relationship between global CBF decline and venous prominence. CBF decline scores emerged as a significant predictor of increased venous visibility, with an OR of 1.690. This implies that for each unit increase in the ASL visual rating scale, indicating a greater CBF decline, the odds of detecting prominent veins increase by 69% (p = 0.004). Age was also a factor, albeit less strongly associated with venous engorgement (OR = 0.953, p = 0.028), suggesting a slight decrease in the likelihood of increased venous visibility with advancing age. Gender did not significantly influence the outcomes (p = 0.829) (Table 3).

**Table 3:** Logistic regression analysis predicting venous prominence This table summarizes the logistic regression analysis examining the influence of ASL stage, age, and gender on the likelihood of increased venous visibility. Significant predictors include the ASL stage, with an odds ratio indicating that each unit increase in the ASL stage is associated with a 1.690 times increase in the odds of observing increased venous visibility. Age also significantly influences venous visibility, albeit to a lesser extent. Gender does not significantly predict venous visibility outcomes. ASL: arterial spin labeling

Variable	Coefficient (B)	Standard error (SE)	Wald	df	Significance (Sig.)	Odds ratio (Exp(B))
ASL stage	0.525	0.182	8.312	1	0.004	1.690
Age	-0.048	0.022	4.825	1	0.028	0.953
Gender	-0.098	0.453	0.047	1	0.829	0.907
Constant	1.664	1.316	1.599	1	0.206	5.279

Inter-rater reliability

The assessment of venous visibility on SWI MRI by two experienced radiologists yielded a high level of agreement, with a Cohen's kappa coefficient of 0.82. This excellent agreement indicates a consistent and reliable evaluation of venous visibility across the study population, underscoring the robustness of the visual assessment methodology employed.

## Discussion

Our study's findings of a significant correlation between global CBF decline and increased visibility of venous structures offer a novel insight into cerebral hemodynamics, contributing to a nuanced understanding of neurovascular health. This section discusses the implications of our findings, their physiological underpinnings, clinical relevance, and directions for future research.

The identification of a positive correlation between global CBF decline and increased venous visibility offers a nuanced extension to existing literature, which has often concentrated on the localized effects of cerebral perfusion changes [11,12]. Unlike previous studies that might have examined specific areas, our analysis suggests that alterations in CBF can have a widespread impact on the venous system, indicating a potential systemic response to changes in perfusion rather than merely isolated regional effects. This broader examination not only aligns with but also deepens our understanding of the brain's vascular architecture, highlighting the complexity and adaptability of cerebral circulation in response to global perfusion changes. By focusing on the entire cerebral venous network, our findings contribute a global perspective that enriches the dialogue on how cerebral hemodynamics adapt to varying physiological and pathological states.

The observed correlation between reduced CBF and increased visibility of venous structures on SWI MRI may reflect complex physiological responses to altered hemodynamics. It is well-established that the brain possesses robust autoregulatory mechanisms designed to maintain oxygenation and nutrient delivery, adjusting vascular resistance and perfusion to meet metabolic demands [13]. This dynamic interplay ensures cerebral homeostasis across a variety of conditions, highlighting the brain's adaptability to changes in arterial inflow [14,15].

However, the hypothesis that venous structures become more visible on imaging as a direct compensatory response to reduced arterial inflow ventures into less charted territories of cerebral hemodynamics. While changes in blood oxygenation levels, venous pressure, and blood volume - all factors that could influence venous visibility on SWI MRI - are indeed potential indicators of the brain's adaptive strategies, the direct linkage of these changes to autoregulatory efforts remains speculative. The visibility of venous structures might be modulated by a range of physiological and pathological conditions, suggesting that observed alterations in SWI MRI could indirectly signal broader hemodynamic adjustments rather than serving as straightforward markers of compensatory mechanisms.

Given this complexity, attributing increased venous visibility uniformly to the brain's autoregulatory adaptation to reduced CBF may oversimplify the underlying physiological processes. It is plausible that under specific pathological or stress conditions, such alterations in venous visibility could indeed reflect broader hemodynamic shifts. This discussion underscores the need for further empirical studies to elucidate the precise nature of the relationship between arterial perfusion changes and venous visibility, particularly how these phenomena are interconnected within the broader context of cerebral autoregulation and vascular compensation.

The observed correlation between global CBF decline and increased venous prominence suggests promising implications for clinical practice, albeit with an understanding of the need for further validation. The non-invasive nature of ASL and SWI MRI to assess changes in cerebral hemodynamics offers a potential tool for improving the detection and monitoring of neurovascular conditions, including stroke, dementia, and chronic vascular diseases. While the integration of these imaging techniques into clinical protocols could potentially enhance diagnostic accuracy and inform treatment strategies, it is important to approach their application with caution. Further research is essential to fully understand the clinical relevance of these findings and to establish robust guidelines for their use in patient care.

A major strength of our study is the use of advanced, non-invasive imaging techniques to explore the relationship between CBF and venous visibility on a global scale. However, limitations include the retrospective design, which may impact the ability to establish causality, and the reliance on visual assessments, introducing potential subjectivity.

Future investigations should focus on longitudinal studies to elucidate the temporal dynamics of CBF decline and venous changes, potentially establishing causality. In addition, the development of quantitative imaging techniques for assessing venous visibility could enhance objectivity and reproducibility. Exploring the relationship between CBF decline and venous visibility in diverse patient populations and across a broader spectrum of neurological conditions would further validate and expand upon our findings.

## Conclusions

Our study reveals a significant global correlation between cerebral blood flow decline and increased venous visibility, highlighting the integral role of advanced MRI techniques in uncovering these relationships. By utilizing ASL and SWI MRI, we offer new insights into the complex interplay of cerebral hemodynamics, providing a foundation for enhanced diagnostic and therapeutic strategies in neurovascular disorders. This research underscores the potential of combining ASL and SWI MRI to improve our understanding of neurovascular health, emphasizing the need for further investigation. Future studies should build on these findings, exploring their broader implications for patient care and advancing the field of neuroimaging in clinical practice.
